# Association of anxiety and depressive symptoms with C-reactive protein in diverse Latinos: Results from the Hispanic Community Health Study/Study of Latinos (HCHS/SOL)

**DOI:** 10.1371/journal.pone.0289833

**Published:** 2023-08-18

**Authors:** Lourdes R. Guerrero, Suzi Hong, Wassim Tarraf, Krista Perreira, Álvaro Camacho, Jordan N. Kohn, Daniel E. Jimenez, Gregory A. Talavera, Linda Gallo, Matthew A. Allison, Sylvia Wassertheil-Smoller, Hector M. González

**Affiliations:** 1 Department of Neurosciences, University of California, San Diego, La Jolla, California, United States of America; 2 Herbert Wertheim School of Public Health and Human Longevity Science, University of California, San Diego, La Jolla, California, United States of America; 3 Department of Psychiatry, University of California, San Diego, La Jolla, California, United States of America; 4 Institute of Gerontology, Wayne State University, Detroit, Michigan, United States of America; 5 Department of Social Medicine, University of North Carolina School of Medicine, Chapel Hill, North Carolina, United States of America; 6 Department of Psychiatry & Behavioral Sciences, University of Miami, Coral Gables, Florida, United States of America; 7 Department of Psychology, San Diego State University, San Diego, La Jolla, California, United States of America; 8 Deparment of Family Medicine, University of California, San Diego, La Jolla, California, United States of America; 9 Department of Epidemiology and Population Health, Albert Einstein College of Medicine, Bronx, New York, United States of America; 10 Shiley Marcos Alzheimer’s Disease Center, University of California, San Diego, La Jolla, California, United States of America; University of Central Florida, UNITED STATES

## Abstract

**Background:**

High sensitivity C-reactive protein (hsCRP) is a marker of systemic inflammation that has been associated with persistent depressive symptoms. Depression and anxiety are frequently associated with a chronic inflammatory state, yet the nature of this relationship has not been rigorously examined in diverse Hispanic/Latino populations. We aimed to study the association of anxiety and depressive symptoms as well as comorbid presentations, with circulating high sensitivity C-reactive protein (hsCRP) levels in a large Latino cohort of diverse heritages. We hypothesized a significant positive associations of both anxiety and depressive symptoms and hsCRP levels and potential variations among the heritage groups.

**Methods:**

Depressive symptoms and anxiety were measured by the Center for Epidemiological Studies Depression Scale (CES-D) and State-Trait Anxiety Inventory (STAI), respectively. Serum hsCRP (hsCRP) levels of 15,448 participants (age 18 to 75 years; 52.3% women) from the Hispanic Community Health Study/Study of Latinos (HCHS/SOL) were measured and categorized based on the established cardiovascular disease (CVD) risk reference values (< 1mg/L, low; 1–<3 mg/L, intermediate; ≥ 3mg/L, high).

**Results:**

Mean CES-D, STAI scores, and hsCRP levels were 7.0 (SD = 5.9), 17.0 (SD = 5.7), and 3.84 (SD = 7.85), respectively. Generalized linear modeling, adjusted for sociodemographic characteristics revealed significant associations between depression (exp(β) = 1.12; p<0.01) and anxiety symptoms (exp(β) = 1.10; p<0.05) with continuous hsCRP levels. For categorical values of hsCRP, one SD increase in CES-D and STAI scores was associated with a 10% and 8% increase in the RRRs of high vs. low hsCRP, respectively. However, these relationships between CES-D or STAI and hsCRP were no longer statistically significant after adjustment for CVD risk factors and medications.

**Conclusion:**

We found modest associations between anxiety and depressive symptoms and systemic inflammation measured by hsCRP among diverse Hispanics/Latinos that did not appreciably differ between heritage groups.

## Background

Nearly 10% of the world population is estimated to live with anxiety and depressive disorders, which are the leading causes of disability worldwide [1]. Anxiety, depression/dysphoria, or comorbid anxiety and depression, have been documented among Hispanics/Latinos [2, 3] and may be associated with a chronic, pro-inflammatory state [4, 5]. Mounting evidence suggests that depressive symptoms and inflammation coexist among both physically asymptomatic individuals and those with chronic conditions such as cardiovascular disease, albeit with mixed findings [6–8]. For example, persons with major depressive disorder (MDD) exhibited higher levels of hsCRP which has been shown to be associated with higher levels of inflammation even after adjusting for body mass index (BMI) [9, 10]. In addition, growing attention is given to associations between anxiety and inflammation as an emerging risk factor for chronic inflammatory conditions such as cardiovascular disease (CVD) [4, 11]. Affective disorders such as generalized anxiety disorder and major depression, as well as elevated depressed mood and inflammation, are at the intersection of multiple biologic pathways [4], and thus are likely related bidirectionally through various mechanisms. For example, pro-inflammatory cytokines in the periphery can directly or indirectly signal the central nervous system (CNS), thereby reducing the bioavailability of monoamine neurotransmitters, like serotonin, by enzymatic breakdown of L-tryptophan, its main amino acid precursor [12]. Importantly, peripheral inflammatory molecules may also play a key pathophysiological role in the progression and development of vascular inflammation and CVD risk, which are elevated among individuals with psychiatric disorders [13, 14]. Furthermore, common psychotherapeutic and pharmacologic treatments for generalized anxiety and depressive disorders have shown to decrease biomarkers of inflammation [5, 7, 15, 16].

In addition to being an established marker of elevated CVD risk, hsCRP has emerged as a stable, reliable biomarker for chronic inflammation and for investigations into the link between depression and inflammation [17], with a meta-analysis demonstrating a nearly 50% increased risk of elevated hsCRP (>3 mg/L) among individuals with depression [18]. Furthermore, a longitudinal, epidemiologic study of anxiety and hsCRP levels in Finland (n = 2688) reported an increased risk of elevated hsCRP in individuals with higher anxiety symptomatology compared to controls (RR = 2.19; 95%CI: 1.08–4.46), and that comorbid anxiety and depression was also associated with higher hsCRP levels (RR = 1.76; 95%CI:1.13–2.74) [16]. Although a recent study of children of HCHS/SOL participants shows a link between depressive symptoms and hsCRP [19], general studies investigating the link between depression and/or anxiety with hsCRP levels in Hispanic/Latino populations are largely lacking.

Previous reports have indicated that Hispanic/Latinos have considerable risks for the development of chronic inflammatory conditions, such as CVD, that are frequently associated with disabling anxiety and depressive states [2, 20, 21]. Interestingly, the prevalence of depressive and anxiety-like symptoms varies widely across Hispanic/Latino backgrounds [3], such that depressive symptoms among individuals of Puerto Rican heritage were nearly twice as high as those of Mexican heritage, as well as by years in the U.S [22]. This finding is consistent with the general understanding that the Hispanic/Latino community is not a monolithic group, and that heritage should be considered and accounted for when examining psychological distress and its comorbidities, such as inflammation [2, 21, 23]. Given the dearth of information on how depressive symptoms and anxiety are associated with chronic inflammatory states with or without physical illness among Latinos, we conducted a cross-sectional study of the association of anxiety and depressive symptoms with inflammation in a large epidemiologic study of Hispanic/Latinos of diverse heritages residing in the United States. We hypothesized that both anxiety and depressive symptoms, as well as co-occuring symptoms of these conditions, would be significantly associated with elevated hsCRP across the diverse groups.

## Methods

### Study population

We conducted an analyses of the baseline data (2008–2011) from the Hispanic Community Health Study/Study of Latinos (HCHS/SOL). Briefly, HCHS/SOL is a multisite cohort study with the objective of identifying risk and protective factors for chronic disease among Hispanic/Latino groups of diverse origin living across the United States. Participants aged 18–74 years (n = 16,415) were recruited using a multistage sampling design from four communities in the United States with large Hispanic/Latino concentrations (San Diego, CA; Bronx, NY; Chicago, IL; Miami, FL). Details of the study rationale, design, and sampling methods were previously published [20, 24]. The Study of Latinos–Investigation of Neurocognitive Aging (SOL-INCA, PI: Gonzalez) is an ancillary study of HCHS/SOL. HCHS/SOL and the SOL-INCA studies were reviewed and approved by the institutional review boards of the University of California, San Diego (UCSD IRB# 171673), and all participating field sites. Written informed consent was obtained from all participants. All data were fully anonymized before we accessed and analyzed them.

Multimodal clinic-based procedures were used to collect and analyze biospecimen (e.g. blood and urine), while standardized questionnaires were used to collect socio-demographic, Hispanic/Latino heritage, and CVD risk factors [20, 24, 25]. For this study, we excluded 172 participants for missing values of the primary outcome (i.e., hsCRP levels), and 795 additional participants with missing values on the primary exposure variables (i.e., CESD or STAI values) (n = 323) or other covariates (n = 472). Our final analytic sample included 15,448 participants from diverse heritage groups including 6,167 Mexicans (39.9%), 2,500 Puerto-Ricans (16.1%), 2,281 Cubans (14.8%), 1,663 Central Americans (10.7%), 1,349 Dominicans (8.7%), 1,017 South Americans (6.6%), and 471 of “other” Latino origins (3.0%). Approximately 20% of the sample was born in the United States.

### C-reactive protein assessment

Serum levels of high sensitivity hsCRP (hsCRP) were measured using a Roche Modular P Chemistry Analyzer using the immunoturbidimetric method (Roche Diagnostics Corporation, IN) from a blood sample drawn via venipuncture during the baseline HCHS/SOL clinical exam. Inter-assay coefficient of variation was reported at <2.5% and intra-assay coefficient of variation was reported at <4.7% [26]. CRP averages did not differ by language of testing (Δmean = -0.12; p = 0.590).

### Measures of depressive and anxiety symptoms

Depressive symptoms were measured with the Andresen version of the 10-item Center for Epidemiological Studies-Depression Scale (CES-D), which has a good predictive accuracy when compared to the full 20-item version with high concordance (k = 0.97) [27] and has been recommended for use with Hispanics/Latinos in English and Spanish [28]. Internal consistency for the current sample was (α = 0.82) and was equivalent for both Spanish and English speakers. Anxiety symptoms were measured via the 10-item Spielberger State Trait Anxiety Inventory (STAI). Respondents were asked to indicate on a scale from 1 (almost never) to 4 (almost always) how often they experienced symptoms related to anxiety over the last week (e.g., I feel nervous and restless). High scores reflect elevated symptoms of anxiety. Internal consistency for the current sample was (α = 0.81). STAI items were equally consistent across language of testing. Chronbach ⍶ = 0.81 for Spanish preferred participants vs. 0.85 for English preferred individuals. Spanish translations of both the CES-D and the STAI were carried out according to recommended translation guidelines (González et al., 2017).

For analyses, both the CES-D-10 and STAI variables were z-scored (X_i_-Mean/SD) to facilitate interpretation across models. In sensitivity models, we also generated and tested a mean z-score derived from both the CES-D and STAI. Additionally, we considered a categorical, nominal, classification of these two measures based on thresholds indicative of clinically-significant depressive and anxiety symptoms (0 = No; 1 = Yes) using cut-off scores CES-D> = 10 and STAI > = 20, respectively (Andresen et al., 1994). Based on these thresholds, individuals were classified as 0 = Low anxiety or depression symptoms, 1 = High anxiety symptoms, 2 = High depressive symptoms, and 3 = High depression and anxiety symptoms.

### Covariates

Hispanic/Latino heritage was determined by self-report, as was information on other socio-demographic characteristics such as age, sex, income (categorized in 4 groups: <$20,000; $20,001–50,000; >$50,000; unreported), level of education (less than high school; high school graduate or equivalent; and >high school) and marital status (single, married/partner, or separated/widowed) [20]. Health and behavioral variables included body mass index (BMI, kg/m^2^), systolic and diastolic blood pressure (BP, mm Hg), smoking status (Yes/No), number of alcoholic drinks per week, and use of antidepressants, antihypertensives, hypoglycemic and lipid lowering medications.

### Statistical methods

Absolute serum hsCRP levels were analyzed as a continuous variable. Additionally, we used categorical grouping of participants into three hsCRP risk groups for CVD based on clinical reference values (< 1mg/L [low risk], 1–<3 mg/L [intermediate risk], and ≥ 3mg/L [elevated risk]) [29, 30].

We generated descriptive statistics to characterize the sociodemographic, health behaviors, and comorbid conditions of the defined population, stratified by hsCRP categories. The estimated means for continuous variables, and percentages for categorical variables are presented in Table 1. We performed survey-adjusted t-tests to test for joint equality of means across heritage groups for continuous measures, and survey-adjusted chi-squared tests to test for significance in categorical variables. Second, we used survey-generalized linear regression models (GLM), assuming a Gaussian distribution and log link function, to examine the associations between the positively skewed hsCRP outcome and each of the exposure variables (depressive and anxiety symptoms), separately (Tables 2 and 3). We also considered alternative GLM models fit using a Poisson rather than a Gaussian distribution; all results remained quantitatively and qualitatively unchanged. In sensitivity analyses, we also modeled the combined continuous (z-scores) and categorical measures of depressive and anxiety symptoms (Supplemental Tables 3 and 4 in S1 File). We used survey multinomial logistic regression to test for associations between categorical hsCRP outcomes (see below) and exposure variables (Tables 2 and 3). In each case, and for each exposure variable, we followed a five-step process beginning with modeling crude associations and incrementally adjusting for 1) age and sex; 2) Hispanic/Latino heritage, education, income and marital status; 3) continous BMI (**kg/m**^**2**^), systolic and diastolic BP (**mm Hg**), smoking status and alcohol use; and 4) aforementioned medication use. Subsequently, to test whether heritage group confers differential risk in the association between the exposure variables and hsCRP, we refitted the age- and sex-adjusted, as well as the fully-adjusted, models to account for and test Hispanic/Latino heritage by depression and anxiety interactions, *respectively*. We conducted an additional set of sensitivity models excluding all participants with self-reported inflammation or swelling based on the following probe (“*Do you have painful inflammation or swelling of your joints that limits your activities*?*”)* and added an additional covariate to control for use of antianxiety agents in the fully adjusted models (Supplemental Tables 5, and 6 and Supplemental Figs 1 and 2 in S1 File).

**Table 1:** Sociodemographic and basic health characteristics and CRP levels of the HCHS/SOL participants by CRP levels.

	CRP Level (mg/L)				
	Low	Intermediate	High	Total	
	% (SE)				
**Sex**					
Female	43.42 (1.12)	47.06 (0.94)	64.71 (1.02)	52.33 (0.58)	p<0.001
**Education**					
Less than high school (HS)	29.80 (1.17)	32.39 (1.08)	33.80 (0.97)	32.15 (0.73)	p = 0.003
HS or Equivalent	28.22 (1.08)	26.86 (0.85)	29.31 (0.98)	28.13 (0.57)	
More than HS	41.98 (1.36)	40.75 (1.11)	36.89 (1.11)	39.72 (0.85)	
**Income**					
<$20,00	37.89 (1.31)	41.49 (1.11)	45.38 (1.12)	41.85 (0.89)	p<0.001
$20,001-	38.74 (1.16)	36.52 (0.96)	35.83 (1.01)	36.91 (0.68)	
> = $50,00	13.68 (1.23)	12.78 (0.96)	9.84 (0.75)	11.99 (0.80)	
Not Reported	9.70 (0.73)	9.21 (0.57)	8.94 (0.58)	9.25 (0.41)	
**Marital Status**					
Single	42.29 (1.07)	30.91 (1.04)	31.03 (1.09)	34.24 (0.68)	p<0.001
Married	45.11 (1.10)	52.46 (1.17)	49.22 (1.18)	49.18 (0.80)	
Separated	12.60 (0.68)	16.63 (0.76)	19.75 (0.85)	16.58 (0.51)	
**Current Smoking**					
Yes	20.33 (0.91)	20.06 (0.87)	22.59 (1.05)	21.04 (0.59)	p = 0.090
**Heritage**					
Dominican	10.39 (0.99)	8.48 (0.71)	10.22 (0.90)	9.65 (0.70)	p<0.001
Central	7.97 (0.75)	7.61 (0.70)	7.10 (0.54)	7.53 (0.56)	
Cuban	19.12 (1.80)	19.96 (1.63)	22.66 (2.09)	20.69 (1.73)	
Mexican	38.91 (2.07)	40.99 (1.85)	33.33 (1.78)	37.65 (1.68)	
Puerto-Rican	13.31 (0.97)	14.29 (0.84)	18.19 (1.12)	15.41 (0.79)	
South American	6.00 (0.59)	4.87 (0.40)	4.13 (0.38)	4.93 (0.32)	
Other	4.30 (0.48)	3.80 (0.44)	4.38 (0.54)	4.15 (0.29)	
**Alcohol Consumption**					
Never	16.75 (1.02)	17.87 (0.92)	20.75 (0.97)	18.58 (0.73)	p<0.001
Former	28.06 (1.05)	28.63 (0.99)	32.23 (1.08)	29.75 (0.68)	
Current	55.19 (1.28)	53.51 (1.13)	47.03 (1.12)	51.67 (0.81)	
**Medication Use**					
Antidepressants	3.35 (0.38)	5.07 (0.40)	6.86 (0.44)	5.21 (0.26)	p<0.001
Anti-hypertensives	7.64 (0.46)	12.13 (0.62)	17.45 (0.75)	12.74 (0.43)	p<0.001
Antidiabetics	5.68 (0.47)	7.34 (0.45)	11.22 (0.62)	8.25 (0.33)	p<0.001
Anti-hyperlipidemics	6.63 (0.45)	9.49 (0.54)	10.82 (0.67)	9.14 (0.36)	p<0.001
	**Mean (SD)**				
**Age (years)**	36.94(14.12)	42.20(14.97)	43.40(15.09)	41.11(15.03)	p<0.001
**BMI (kg/m** ^**2**^ **)**	25.65(4.07)	28.97(4.81)	32.74(6.87)	29.36(6.12)	p<0.001
**Alcoholic Drinks (week)**	3.04(6.38)	3.06(6.92)	2.37(6.12)	2.81(6.51)	p<0.001
**Average Systolic (mm Hg)**	116.70(14.56)	120.76(17.00)	121.70(19.31)	119.92(17.27)	p<0.001
**Average Diastolic (mm Hg)**	68.70(9.61)	72.67(10.81)	74.50(11.32)	72.18(10.90)	p<0.001
**CESD-10 (0–30)**	6.42(5.12)	6.72(5.79)	7.68(6.60)	6.98(5.92)	p<0.001
**STAI-10 (10–40)**	16.64(5.06)	16.74(5.67)	17.54(6.18)	17.00(5.69)	p<0.001

**Notes:** BMI: Body Mass Index; CRP: C-reactive protein; CES-D: Center for Epidemiological Studies Depression Scale; STAI: Spielberger Trait-State Anxiety Inventory; BP: Blood Pressure; HS: High School. All chi-squared tests (for categorical) and t-tests (for continuous measures are significant at p<0.01.

**Table 2:** Association of standardized depressive symptoms (in SD units) with CRP^a^^,^^b^ levels among Hispanics/Latinos of diverse backgrounds.

	Crude	Age & Sex Adj	Dem Adj	Risk Adj	Med Adj
	exp(β) [95% CI]	exp(β) [95% CI]	exp(β) [95% CI]	exp(β) [95% CI]	exp(β) [95% CI]
**CRP (Continuous Metric)** ^a^					
Standardized CESD-10	1.18***[1.10;1.26]	1.13**[1.05;1.21]	1.12**[1.03;1.21]	1.05[0.99;1.10]	1.05[0.99;1.11]
**CRP (3-Category, Ref: Low)** ^b^					
Intermediate	1.06[1.00;1.12]	1.02[0.96;1.09]	1.02[0.96;1.09]	0.99[0.93;1.06]	0.99[0.93;1.05]
High	1.24***[1.17;1.31]	1.13***[1.07;1.21]	1.10**[1.03;1.17]	1.01[0.94;1.08]	1.00[0.93;1.08]

^*a*^ estimates based on survey generalized linear regression models assuming a Gaussian distribution and a log link function.

^b^ estimates based on survey multinomial logit models.

CRP: C-Reactive Protein. CESD-10: Center for Epidemiological Studies-Depression Scale.

*Crude*: Unadjusted.

*Age & Sex Adj*: Crude + age, sex

*Dem Adj*: Age & Sex Adj + Latino background, education, income, marital status.

*Risk Adj*: Dem Adj *+* BMI, systolic BP, diastolic BP, current smoker, alcohol drinks/week.

*Med Adj*: Risk Adj*+* antidepressants, antihypertensives, hypoglycemic, lipid lowering medications.

***p<0.001

**p<0.01

*p<0.*05*

**Table 3:** Association of standardized anxiety symptoms (in SD units) with CRP^a^^,^^b^ levels among Hispanics/Latinos of diverse backgrounds.

	Crude	Age & Sex Adj	Dem Adj	Risk Adj	Med Adj
	exp(β) [95% CI]	exp(β) [95% CI]	exp(β) [95% CI]	exp(β) [95% CI]	exp(β) [95% CI]
**CRP (Continuous Metric)** ^a^					
Standardized STAI-10	1.15***[1.06;1.25]	1.11**[1.03;1.20]	1.10*[1.02;1.19]	1.04[0.98;1.09]	1.04[0.98;1.09]
**CRP (3-Category, Ref: Low)** ^b^					
Intermediate	1.02[0.96;1.08]	1.02[0.96;1.08]	1.01[0.95;1.08]	0.98[0.92;1.04]	0.97[0.91;1.04]
High	1.17***[1.11;1.24]	1.12***[1.05;1.18]	1.08**[1.02;1.15]	0.99[0.92;1.07]	0.99[0.92;1.06]

^*a*^ estimates based on survey generalized linear regression models assuming a Gaussian distribution and a log link function.

^b^ estimates based on survey multinomial logit models.

CRP: C-Reactive Protein. STAI-10: Spielberger Trait Anxiety Inventory

*Crude*: unadjusted.

*Age & Sex Adj*: Crude + age, sex

*Dem Adj*: Age & Sex Adj + Latino background, education, income, marital status.

*Risk Adj*: Dem Adj *+* BMI, systolic BP, diastolic BP, current smoker, alcohol drinks/week.

*Med Adj*: Risk Adj*+* antidepressants, antihypertensives, hypoglycemic, lipid lowering medications.

***p<0.001

**p<0.01

*p<0.05

**Fig 1:**
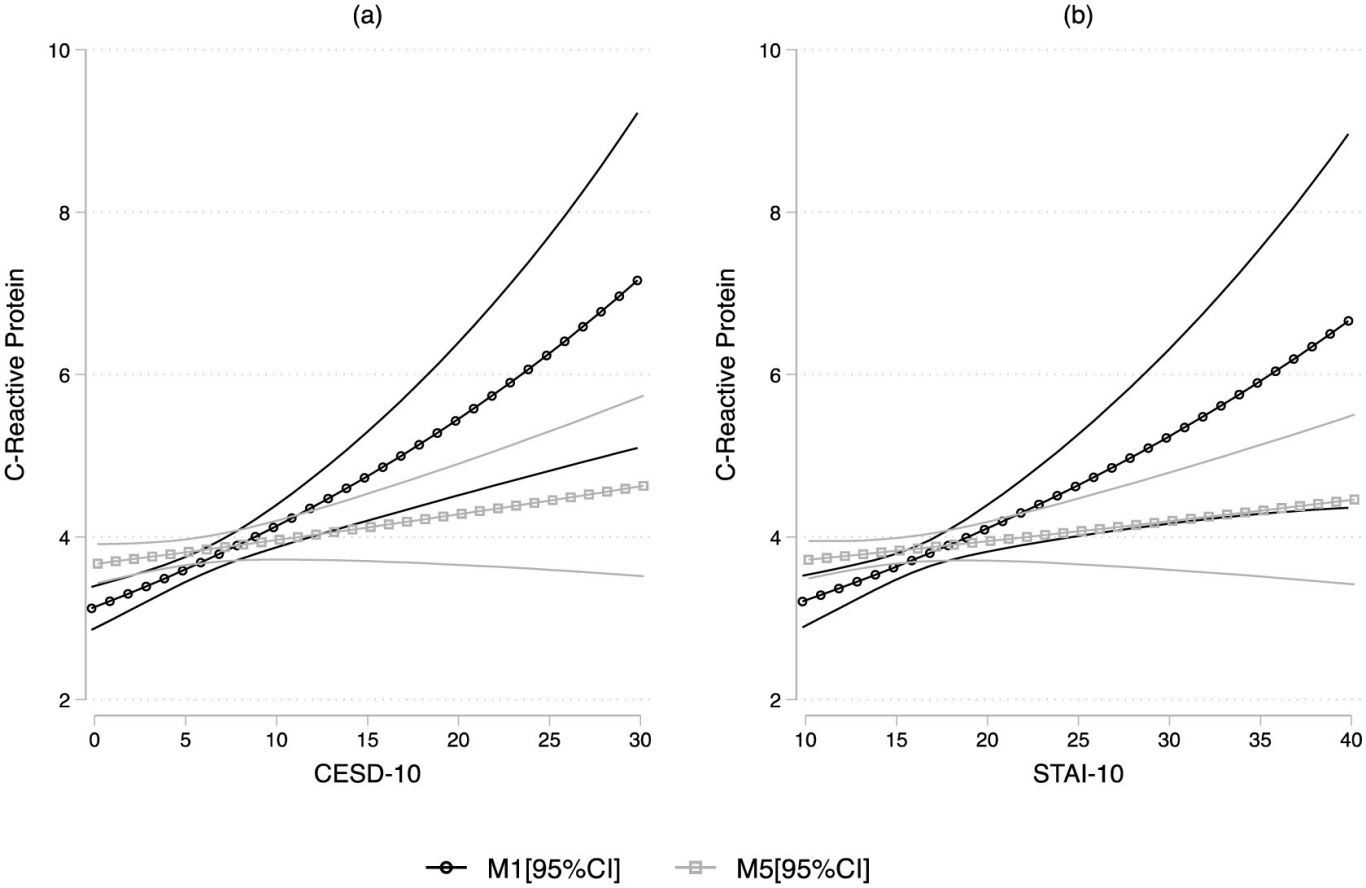
Estimated marginal means of the hsCRP levels. The increases in circulating hsCRP levels over the range of (a) CESD-10 (Center for Epidemiologic Study Depression scale) and (b) STAI-10 (Spielberger Trait Anxiety Inventory) scores and their 95% confidence intervals are plotted from the crude (M1) and fully-adjusted models (M5), controlling for sociodemographic (age, sex, Latino background, education, marital status, and income), health and behavior (BMI, systolic and diastolic BP, current smoker, alcohol drinks/week), and medications (antidepressants, antihypertensives, hypoglycemic, lipid lowering medications).

**Fig 2:**
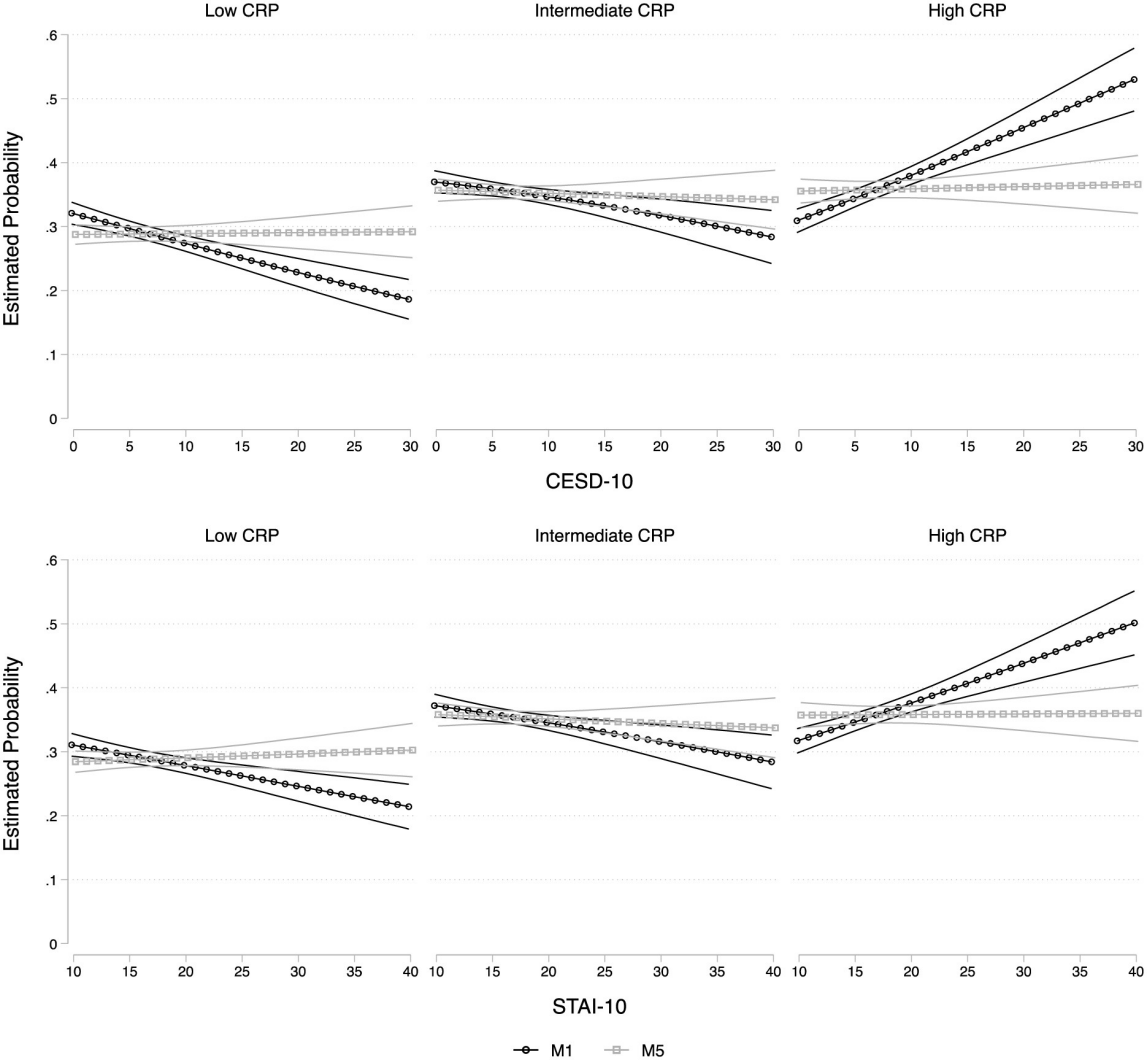
Estimated marginal probabilities for the hsCRP risk categories. The estimated probabilities for each hsCRP risk group (low, medium, and high) over the range of CESD-10 (Center for Epidemiologic Study Depressionx; top) and STAI (Spielberger Trait Anxiety Inventory; bottom) scores and their 95% confidence intervals are plotted from the crude (M1) and fully-adjusted models (M5), controlling for sociodemographic (age, sex, Latino background, education, marital status, and income), health and behavior (BMI, systolic and diastolic BP, current smoker, alcohol drinks/week), and medications (antidepressants, antihypertensives, hypoglycemic, lipid lowering medications).

For the continuous hsCRP measure, we report estimates of the exponentiated Beta coefficients and their 95% confidence intervals for ease of interpretation of the original scale. Reporting the exponentiated coefficients allows for a % interpretation in unit increases in the mean of the outcome relative to a unit increase in the predictors. For multinomial logit models, we set low hsCRP as the reference outcome category and reported the estimated relative risk ratios. (RRRs) and their 95% confidence intervals. To facilitate the interpretation of our estimates, we computed and plotted the marginal means (Fig 1a) and probabilities (Fig 2a) of the hsCRP outcomes resulting from the crude and fully adjusted models, and their 95% confidence intervals, over the range of the CES-D and STAI values. In sensitivity models, we also considered a four-risk group classification for hsCRP (< 1mg/L [low risk], 1–<3 mg/L [intermediate risk], and 3-<5 mg/L [elevated risk], and ≥ 5mg/L [high risk], given the literature on the association between high hsCRP levels (≥ 5mg/L) and depression (Supplemental Tables 2–4 in S1 File) [7].

All analyses were conducted using Stata version 17.0 (StataCorp, College Station, TX) using functionalities designed to account for the complex design of the HCHS/SOL including clustering, stratification, and adjustment for sampling probability and non-response [25]. Appropriate survey subpopulation analysis techniques and HCHS/SOL weights were used to generate estimates and inferential statistics [25].

## Results

Descriptive characteristics of the study population are presented by hsCRP groups (Table 1). The mean cohort age was 41 years, and approximately half of the population was female. Close to one-third of the target population reported less than high school education, slightly more than 40% reported an income level at or less than $20,000, and about 50% were married. About 20% of the sample reported smoking cigarettes currently. Thirty-seven percent (37.1%) of the cohort was overweight or obese (39.7) with a mean BMI of 29.4 kg/m^2^, though on average, had normal BP and reported consuming approximately 3 alcoholic drinks per week.

Participants who were excluded due to missing hsCRP values showed similar demographic (e.g., sex, age, education, and income) characteristics to those included and were not statistically distinct in terms of mean CES-D or STAI scores. In addition, participants excluded due to missing CES-D or STAI data did not differ significantly in their sex, age, education, or income characteristics compared to their included counterparts.

The proportion of participants exhibiting low (<1mg/L), intermediate (1-<3mg/L), and elevated (≥ 3mg/L) hsCRP levels was 28.8%, 35.3%, and 35.8%, respectively. We found significant variations in CES-D scores by hsCRP groups (Table 2). Additional demographic, CVD, and behavioral risk factors, and medication use charactersitics by hsCRP levels are presented in Table 1.

### Depressive symptoms and inflammation

#### CES-D and hsCRP concentrations (Table 2)

Initial analyses indicated a significant association between CES-D scores and hsCRP levels, such that there was an 18% increase (95% CI: 1.10, 1.26; 13% after adjustment for age and sex) in hsCRP levels for each 1-SD increase in the CES-D scores (Fig 1a). The association between CES-D and hsCRP remained significant, but slightly attenuated, after adjusting for the socio-demographic factors (age, sex, Hispanic/Latino background, education, income, and marital status). Following additional adjustment for cardiovascular (i.e., BMI and BP) and behavioral (i.e., smoking and alcohol consumption) risk factors, the hsCRP-CES-D association was further attenuated and was no longer significant at α = 0.05. Adjustment for medications (i.e., antidepressants, antihypertensives, antidiabetic, and lipid lowering medications) resulted in no significant change to the model. Sensitivity models fit *post hoc*, by including each CVD measure independently in the model, suggest that each of the measures had an attenutating effect on the association between CES-D and hsCRP, with the attenuation being most pronounced for BMI.

#### CES-D and hsCRP in categories (Table 2)

Similar results emerged from the multinomial logistic analyses using hsCRP categories, with some notable additions. Using low hsCRP levels (<1 mg/L) as the reference category, and for each SD increase in CES-D score, there was a 24% increase in the RRRs of high (≥3mg/L) vs. low hsCRP classification (p<0.001). There was no significant difference between low and middle hsCRP categories in its association with CES-D scores. As with the continuous hsCRP level findings, the RRRs were attenuated after adjusting for age, sex, and other socio-demographic factors, and further attenuated to non-significance by additional adjustment for behavioral, cardiovascular, and medication confounders. Fig 2 displays the estimated probabilities for each hsCRP group (low, intermediate, and high) over the range of CES-D scores and their 95% confidence intervals.

### Anxiety symptoms and inflammation

#### STAI scores and hsCRP concentrations (Table 3)

Similar to depressive symptoms, univariable analyses indicated a significant association between STAI scores and hsCRP levels such that there was a 15% increase (95% CI: 1.06, 1.25; 11% after adjustment for age and sex) in hsCRP levels for each SD increase in STAI score (Fig 1b). The association between STAI and hsCRP persisted after adjustment for additional socio-demographic factors, but was no longer significant after adjustment for behavioral and cardiovascular risk factor, and medication use. Sensitivity models fit *post hoc*, by including each CVD measure independently in the model, were similar to those found for the CES-D.

#### STAI scores and hsCRP in categories (Table 3)

As with depressive symptoms, the association between hsCRP and anxiety was restricted to the high hsCRP risk group (relative to low risk). Specifically, a 1-SD increase in STAI score was associated with a 17% increased RRRs of high vs. low hsCRP classification (Fig 2). RRRs were attenuated with an adjustment for age, sex, and other socio-demographic factors, and were no longer significant after additional adjustments for behavioral and medication covariates.

#### Latino heritage exposure interactions

Although there were significant heritage-group based differences in hsCRP, CES-D, and STAI levels, these did not significantly moderate the observed associations between hsCRP-CES-D or hsCRP-STAI (Supplemental Table 1 in S1 File).

#### Sensitivity analyses for higher hsCRP levels and comorbid depression and anxiety symptoms

Results from sensitivity models were qualitatively consistent with the main findings presented above. First, estimates for the four hsCRP risk groups, generated by further separating individuals with hsCRP ≥5 mg/L, showed no differentiation in the magnitude or significance of the coefficients between the elevated (3-<5 mg/L) and high (5+ mg/L) risk groups relative to the low (< 1mg/L) hsCRP group. This was consistent for both the CES-D and STAI (Supplemental Table 2 in S1 File). Second, the magnitude and significance of the combined average z-scores for CES-D and STAI in predicting hsCRP levels were consistent with estimates generated from models using these indicators independently (Supplemental Table 3 in S1 File). More specifically, higher mixed-symptom scores were associated with greater hsCRP levels in socio-demographic adjusted models, but the association was attenuated to non-significance after adjusting for behavioral, CVD, and medication factors. These results were concordant for both continuous and categorical operationalizations of hsCRP. Lastly, ‘anxious-depression’ which was operationalized categorically for comorbid high anxiety and depression (vs. anxiety or depression only), was associated with higher hsCRP values (Supplemental Table 4 in S1 File). This was consistent for both continuous and categorical operationalizations of hsCRP.

Sensitivity models excluding participants with self-reported inflammation of joint swelling. Results from these models were qualitatively undifferentiated from the primary findings. The estimates and their confidence intervals are reported in Supplemental Tables 5 and 6 (S1 File), and marginal means (for continuous CRP) and probability (for categorical CRP) plots are included in Supplemental Figures 1 and 2 (S1 File).

## Discussion

Our large, population-based, cross-sectional study of Hispanics/Latinos from diverse heritages revealed modest associations between anxiety and depressive symptoms and systemic inflammation measured by hsCRP, and to our knowledge, is the first study to examine both anxiety and depressive symptoms in association with inflammation across a large cohort of diverse Hispanic/Latino adults. Our findings extend the existing clinical research findings to the population-level that depressed mood was associated with systemic inflammation [4, 31–33]. Additionally, our findings add to the literature by describing anxiety-inflammation associations and the potential role of comorbid anxiety and depressive symptoms with inflammation as a risk factor for the development of chronic inflammatory conditions, including cardiovascular disease [5, 11, 34–39].

The association of anxiety and/or depressive symptoms with hsCRP was markedly attenuated by cardiovascular and behavioral risk factors, including BMI which is highly related to inflammation [40] and a risk factor for the development of cardiovascular and metabolic disorders [41]. Relationships among depressive symptoms, obesity, and inflammation have been previously demonstrated [19, 42], and it was not surprising that BMI weakened the depression-hsCRP associations. Yet, to our knowledge, this is the first report that such findings are evident in diverse Hispanic/Latino adult populations. Existing literature suggests that anxiety and depressive states have worse prognoses among individuals with metabolic disorders, such as type 2 diabetes (T2D) [43]. How symptoms of anxiety and depression are related to chronic inflammatory states and cardiometabolic disorders, such as metabolic syndrome and T2D, and how these differ between Latino heritage groups warrants further examination, as Hispanics/Latinos are at relatively high risk for T2D [44].

Few studies have examined associations between elevated anxiety/depression symptoms and inflammation among diverse Hispanic/Latinos. In this study, we did not find notable differences between depression and/or anxiety with inflammation among the different Hispanic/Latino heritage groups. Previous studies from the HCHS/SOL reported that individuals of Puerto Rican heritage had the highest rate of comorbid anxiety and depression symptoms, and the highest number of risk factors, such as obesity, for chronic inflammatory conditions relative to other Hispanics/Latinos [2, 20, 21]. Another HCHS/SOL study reported prevalent anxiety and depression was associated with diabetes, particularly those of Mexican heritage [45]. Although the Puerto Rican group had the highest hsCRP levels, reporting slightly higher levels of anxiety and depressive symptoms, and living in the United States [46], the association of anxiety and depression symptoms with inflammation in our study did not markedly differ between them and the other Hispanic/Latino heritage groups. Hispanics/Latinos face various psychosocial stressors, including discrimination, poverty and immigration concerns, which may be important mediators between depression and anxiety symptoms and markers of inflammation [22, 47, 48]. Additional information is also needed to understand protective factors among these groups. Information on the use of antidepressants and anti-anxiety medications could be an influencing factor, but this data was not available for analysis.

Our study has a few limitations for consideration. First, this was a cross-sectional observational study and our findings can not be portrayed as causal. The measures of depression and anxiety used were symptom-based only and not diagnostic. Thus, our findings can not be fully extended to clinical populations. Second, we did not consider other inflammatory biomarkers (e.g., interleukin-6), which may have offered an opportunity to characterize individuals’ inflammatory state more comprehensively [4, 5, 26]. We also recognize that there may be unaccounted for differences among our study sample due to the varying geographic regions they represent, and due to sexual gender minority status which is information we did not have. Furthermore, analysis of immigrant status (U.S. born, born in Puerto Rico, born in other countries), age of migration, years of living in the U.S. and neighborhood characteristics [49, 50] could also be important moderators, mediators or protective factors to consider in studies of inflammation among Latino/Hispanic populations.

## Conclusion

In conclusion, our study extends existing research regarding links between anxiety and depression, and inflammation to diverse Hispanics/Latinos, an important and growing population. Various studies have shown that treating systemic inflammation using anti-inflammatory medication improved depressive symptoms among treatment-resistant patients with depression and anxiety with elevated inflammation levels [12, 51, 52]. Thus, there is a need to explore other markers of inflammation as they relate to mental health symptoms among Hispanic/Latino groups and how additional psychosocial stressors, like caregiver status and/or perceptions of racism, may influence those relationships. Having a better understanding of inflammatory markers among diverse Hispanic/Latinos could pave the way for the development of appropriate therapeutic interventions for varying heritage groups.

## Supporting information

S1 File Contains all the supplementary tables and supplementary figures. (DOCX)
