# Supplementary material for: Association of anxiety and depressive symptoms with C-reactive protein in diverse Latinos: Results from the Hispanic Community Health Study/Study of Latinos (HCHS/SOL)
Source: PLoS One. 2023 Aug 18;18(8):e0289833. doi: 10.1371/journal.pone.0289833 (PMC10437793; doi:10.1371/journal.pone.0289833)
Supplement: S1 File — (DOCX) [file pone.0289833.s001.docx]

**Supplemental Table 1**. Survey adjusted F-Tests for the interactions between CESD-10 and STAI-10 and Latino background. Results are derived from Generalized Linear Models (for continuous CRP) and multinomial logit model (for categorical CRP).

|  |  | **Age, Sex Adjusted** | | | | **Fully Adjusted** | | | |
| --- | --- | --- | --- | --- | --- | --- | --- | --- | --- |
|  |  | **F-Test** | | **p-value** | | **F-Test** | | **p-value** | |
| **Depression (CESD-10)** | |  |  | |  | |  | |  |
|  | **Continuous CRP** | F( 6,639) = 1.91 | | 0.0769 | | F(6,639) = 1.17 | | 0.3182 |  |
|  | **Categorical CRP (Ref: Low)** |  | |  | |  | |  | |
|  | Intermediate | F(6, 639) = 0.63 | | 0.7056 | | F(6,639) = 0.73 | | 0.6271 | |
|  | High | F(6,639) = 0.46 | | 0.8378 | | F(6,639) = 0.65 | | 0.6928 | |
|  |  |  | |  | |  | |  | |
| **Anxiety (STAI-10)** | |  | |  | |  | |  | |
|  | **Continuous CRP** | F(6639) = 1.11 | | 0.3573 | | F(6,639) = 1.17 | | 0.3212 | |
|  | **Categorical CRP (Ref: Low)** |  | |  | |  | |  | |
|  | Intermediate | F(6,639) = 0.84 | | 0.5409 | | F(6,639) = 0.89 | | 0.5021 | |
|  | High | F( 6,639) = 0.51 | | 0.8024 | | F(6,639) = 0.52 | | 0.791 | |

CRP: C-Reactive Protein; CESD-10: Center for Epidemiologic Study Depression scale; STAI-10: Spielberger Trait Anxiety Inventory

**Supplemental Table 2.** Association of standardized anxiety and depressive symptoms (in SD units) with 4-category CRP^a^ levels among Latinos of diverse backgrounds

|  | **Crude** | **Age & Sex Adj** | **Dem Adj** | **Risk Adj** | **Med Adj** |
| --- | --- | --- | --- | --- | --- |
|  | **exp(β) [95% CI]** | **exp(β) [95% CI]** | **exp(β) [95% CI]** | **exp(β) [95% CI]** | **exp(β) [95% CI]** |
| **CESD-10 (Ref: Low CRP)^a^** |  |  |  |  |  |
| Intermediate | 1.06[1.00;1.12] | 1.02[0.96;1.09] | 1.02[0.96;1.09] | 0.99[0.93;1.06] | 0.99[0.93;1.05] |
| High | 1.21***[1.12;1.30] | 1.13**[1.04;1.21] | 1.10*[1.02;1.19] | 1.03[0.95;1.11] | 1.03[0.95;1.11] |
| Very High | 1.26***[1.18;1.35] | 1.14***[1.06;1.22] | 1.09*[1.02;1.18] | 0.99[0.91;1.08] | 0.98[0.90;1.08] |
|  |  |  |  |  |  |
| **STAI-10 (Ref: Low CRP)^a^** |  |  |  |  |  |
| Intermediate | 1.02[0.96;1.08] | 1.02[0.96;1.08] | 1.01[0.95;1.08] | 0.98[0.92;1.04] | 0.97[0.91;1.04] |
| High | 1.16***[1.08;1.24] | 1.12***[1.05;1.20] | 1.10**[1.03;1.18] | 1.03[0.95;1.10] | 1.02[0.95;1.11] |
| Very High | 1.18***[1.10;1.26] | 1.11**[1.03;1.19] | 1.07[0.99;1.15] | 0.96[0.88;1.05] | 0.95[0.87;1.04] |

^a^ estimates based on survey multinomial logit models (relative risk ratios reported; RRRs).

CRP: C-Reactive Protein. CESD-10: Center for Epidemiological Studies-Depression Scale. STAI-10: Spielberger Trait Anxiety Inventory.

*Crude*: Unadjusted.

*Age & Sex Adj*: Crude + age, sex

*Dem Adj:* Age & Sex Adj + Latino background, education, income, marital status.

*Risk Adj:* Dem Adj *+* BMI, systolic BP, diastolic BP, current smoker, alcohol drinks/week.

*Med Adj:* Risk Adj*+* antidepressants, antihypertensives, hypoglycemic, lipid lowering medications.

***p<0.001

**p<0.01

**p<0.05*

**Supplemental Table 3**. Association of standardized mixed anxiety and depressive symptoms (in SD units) with CRP^a,b^ levels among Latinos of diverse backgrounds.

|  | **Crude** | **Age & Sex Adj** | **Dem Adj** | **Risk Adj** | **Med Adj** |
| --- | --- | --- | --- | --- | --- |
|  | **exp(β) [95% CI]** | **exp(β) [95% CI]** | **exp(β) [95% CI]** | **exp(β) [95% CI]** | **exp(β) [95% CI]** |
| **CRP (Continuous Measure) ^a^** |  |  |  |  |  |
| Standardized Mixed CESD-10 & STAI-10 | 1.19***[1.09;1.31] | 1.14**[1.05;1.25] | 1.13*[1.03;1.24] | 1.05[0.99;1.11] | 1.05[0.98;1.12] |
| **CRP (3-Category, Ref: Low) ^b^** |  |  |  |  |  |
| Intermediate | 1.04[0.98;1.11] | 1.02[0.96;1.09] | 1.02[0.95;1.09] | 0.98[0.91;1.05] | 0.98[0.91;1.05] |
| High | 1.24***[1.17;1.32] | 1.15***[1.07;1.22] | 1.11**[1.04;1.18] | 1.00[0.92;1.08] | 0.99[0.92;1.08] |
| **CRP (4-Category, Ref: Low) ^b^** |  |  |  |  |  |
| Intermediate | 1.04[0.98;1.11] | 1.02[0.96;1.09] | 1.02[0.95;1.09] | 0.98[0.91;1.05] | 0.98[0.91;1.05] |
| High | 1.22***[1.12;1.31] | 1.15***[1.06;1.24] | 1.12**[1.04;1.21] | 1.03[0.95;1.12] | 1.03[0.94;1.12] |
| Very High | 1.26***[1.17;1.35] | 1.15***[1.06;1.24] | 1.10*[1.01;1.19] | 0.97[0.88;1.07] | 0.96[0.87;1.06] |

*^a^* estimates based on survey generalized linear regression models assuming a Gaussian distribution and a log link function.

^b^ estimates based on survey multinomial logit models (relative risk ratios reported; RRRs).

CRP: C-Reactive Protein. STAI-10: Spielberger Trait Anxiety Inventory. CESD-10: Center for Epidemiological Studies-Depression Scale.

*Crude*: Unadjusted.

*Age & Sex Adj*: Crude + age, sex

*Dem Adj:* Age & Sex Adj + Latino background, education, income, marital status.

*Risk Adj:* Dem Adj *+* BMI, systolic BP, diastolic BP, current smoker, alcohol drinks/week.

*Med Adj:* Risk Adj*+* antidepressants, antihypertensives, hypoglycemic, lipid lowering medications.

***p<0.001 **p<0.01 *p<0.05

**Supplemental Table 4**. Association of grouped anxiety and depressive symptoms with CRP^a,b^ levels among Latinos of diverse backgrounds.

|  | **Crude** | **Age & Sex Adj** | **Dem Adj** | **Risk Adj** | **Med Adj** |
| --- | --- | --- | --- | --- | --- |
|  | **exp(β) [95% CI]** | **exp(β) [95% CI]** | **exp(β) [95% CI]** | **exp(β) [95% CI]** | **exp(β) [95% CI]** |
| **CRP (Continuous Measure, Ref: No anxiety/depression)^a^** | | | | | |
| Anxiety only | 0.96[0.86;1.07] | 0.95[0.86;1.06] | 0.94[0.84;1.05] | 0.97[0.86;1.08] | 0.97[0.86;1.08] |
| Depression only | 1.22*[1.05;1.43] | 1.16[0.98;1.37] | 1.16[0.91;1.47] | 1.07[0.91;1.25] | 1.07[0.90;1.27] |
| Anxiety and depression | 1.37***[1.18;1.59] | 1.25**[1.08;1.46] | 1.22*[1.04;1.41] | 1.05[0.94;1.18] | 1.05[0.93;1.19] |
|  | | | | | |
| **CRP (3-Category, Ref: Low)^b^** | | | | | |
| **Intermediate (Ref: No anxiety/depression)** | | | | | |
| Anxiety only | 0.92[0.76;1.13] | 0.97[0.79;1.19] | 0.95[0.78;1.17] | 0.94[0.76;1.16] | 0.94[0.76;1.16] |
| Depression only | 1.18[0.95;1.45] | 1.07[0.86;1.32] | 1.07[0.86;1.34] | 1.03[0.83;1.28] | 1.03[0.83;1.28] |
| Anxiety and depression | 1.19*[1.00;1.41] | 1.12[0.94;1.33] | 1.11[0.93;1.33] | 1.00[0.83;1.19] | 0.99[0.83;1.19] |
| **High (Ref: No anxiety/depression)^b^** | | | | | |
| Anxiety only | 0.87[0.72;1.04] | 0.87[0.72;1.05] | 0.84[0.70;1.02] | 0.79*[0.64;0.98] | 0.79*[0.63;0.98] |
| Depression only | 1.40**[1.14;1.73] | 1.16[0.94;1.45] | 1.13[0.91;1.41] | 1.02[0.81;1.28] | 1.02[0.81;1.28] |
| Anxiety and depression | 1.70***[1.44;2.00] | 1.41***[1.20;1.66] | 1.30**[1.10;1.53] | 1.02[0.85;1.22] | 1.01[0.84;1.22] |
|  | | | | | |
| **CRP (4-Category, Ref: Low)^b^** | | | | | |
| **Intermediate (Ref: No anxiety/depression)** | | | | | |
| Anxiety only | 0.92[0.76;1.13] | 0.97[0.79;1.19] | 0.95[0.78;1.17] | 0.94[0.76;1.16] | 0.94[0.76;1.16] |
| Depression only | 1.18[0.95;1.45] | 1.07[0.86;1.32] | 1.07[0.86;1.34] | 1.03[0.83;1.28] | 1.03[0.83;1.28] |
| Anxiety and depression | 1.19*[1.00;1.41] | 1.12[0.94;1.33] | 1.11[0.93;1.33] | 0.99[0.83;1.19] | 0.99[0.82;1.19] |
| **High (Ref: No anxiety/depression)^b^** | | | | | |
| Anxiety only | 0.83[0.67;1.03] | 0.85[0.68;1.06] | 0.83[0.66;1.04] | 0.79[0.62;1.01] | 0.79[0.62;1.01] |
| Depression only | 1.42*[1.09;1.87] | 1.21[0.92;1.60] | 1.19[0.90;1.57] | 1.09[0.83;1.43] | 1.09[0.83;1.44] |
| Anxiety and depression | 1.64***[1.33;2.01] | 1.41***[1.15;1.73] | 1.34**[1.10;1.64] | 1.09[0.88;1.35] | 1.09[0.88;1.36] |
| **Very High (Ref: No anxiety/depression)^b^** | | | | | |
| Anxiety only | 0.89[0.72;1.11] | 0.89[0.71;1.11] | 0.85[0.68;1.07] | 0.79[0.61;1.02] | 0.78[0.60;1.02] |
| Depression only | 1.39**[1.10;1.74] | 1.13[0.90;1.43] | 1.09[0.87;1.38] | 0.96[0.74;1.23] | 0.95[0.74;1.22] |
| Anxiety and depression | 1.75***[1.45;2.11] | 1.41***[1.16;1.70] | 1.27*[1.05;1.53] | 0.96[0.78;1.19] | 0.94[0.76;1.17] |

*^a^* estimates based on survey generalized linear regression models assuming a Gaussian distribution and a log link function.

^b^ estimates based on survey multinomial logit models (relative risk ratios reported; RRRs).

CRP: C-Reactive Protein

Classification of CES-D and STAI based on thresholds indicative of high depressive and anxiety symptoms (0=No; 1=Yes) using CES-D>=10 and STAI >=20, respectively. Based on these thresholds’ individuals were classified as 0=No anxiety or depression, 1=High anxiety symptoms (anxiety only), 2=High depressive symptoms (depression only), and 3=High depression and anxiety symptoms (anxiety and depression).

**Supp Table 5.** Association of standardized Depressive symptoms (in SD units) with CRP^a,b^ levels among Hispanics/Latinos of diverse backgrounds. Models exclude individuals with self-reported inflammation or swelling (“*Do you have painful inflammation or swelling of your joints that limits your activities?”)*

|  | **Crude** | **Age & Sex Adj** | **Dem Adj** | **Risk Adj** | **Med Adj** |
| --- | --- | --- | --- | --- | --- |
|  | **exp(β) [95% CI]** | **exp(β) [95% CI]** | **exp(β) [95% CI]** | **exp(β) [95% CI]** | **exp(β) [95% CI]** |
| **CRP (Continuous Metric) ^a^** |  |  |  |  |  |
| Standardized CESD-10 | 1.16**[1.06;1.27] | 1.12*[1.02;1.22] | 1.11[1.00;1.24] | 1.05[0.97;1.13] | 1.05[0.97;1.13] |
| **CRP (3-Category, Ref: Low) ^b^** |  |  |  |  |  |
| Intermediate | 1.05[0.98;1.11] | 1.03[0.96;1.09] | 1.03[0.96;1.10] | 1.00[0.94;1.07] | 1.00[0.94;1.07] |
| High | 1.17***[1.10;1.25] | 1.10**[1.03;1.17] | 1.07[1.00;1.14] | 1.01[0.93;1.09] | 1.00[0.93;1.08] |

*^a^* estimates based on survey generalized linear regression models assuming a Gaussian distribution and a log link function.

^b^ estimates based on survey multinomial logit models (relative risk ratios reported; RRRs).

CRP: C-Reactive Protein. CESD-10: Center for Epidemiological Studies-Depression Scale.

*Crude*: Unadjusted.

*Age & Sex Adj*: Crude + age, sex

*Dem Adj:* Age & Sex Adj + Latino background, education, income, marital status.

*Risk Adj:* Dem Adj *+* BMI, systolic BP, diastolic BP, current smoker, alcohol drinks/week.

*Med Adj:* Risk Adj*+* antidepressants, antianxiety agents, antihypertensives, hypoglycemic, lipid lowering medications.

***p<0.001

**p<0.01

**p<0.05*

**Supp Table 6**. Association of standardized Anxiety symptoms (in SD units) with CRP^a,b^ levels among Hispanics/Latinos of diverse backgrounds. Models exclude individuals with self-reported inflammation or swelling (“*Do you have painful inflammation or swelling of your joints that limits your activities?”)*

|  | **Crude** | **Age & Sex Adj** | **Dem Adj** | **Risk Adj** | **Med Adj** |
| --- | --- | --- | --- | --- | --- |
|  | **exp(β) [95% CI]** | **exp(β) [95% CI]** | **exp(β) [95% CI]** | **exp(β) [95% CI]** | **exp(β) [95% CI]** |
| **CRP (Continuous Metric) ^a^** |  |  |  |  |  |
| Standardized STAI-10 | 1.14*[1.03;1.27] | 1.11*[1.00;1.22] | 1.10[0.99;1.23] | 1.05[0.97;1.13] | 1.05[0.97;1.13] |
| **CRP (3-Category, Ref: Low) ^b^** |  |  |  |  |  |
| Intermediate | 1.01[0.96;1.08] | 1.02[0.96;1.08] | 1.02[0.95;1.08] | 0.99[0.93;1.05] | 0.99[0.92;1.05] |
| High | 1.12***[1.06;1.20] | 1.08*[1.02;1.15] | 1.05[0.99;1.12] | 1.00[0.92;1.07] | 0.99[0.92;1.07] |

*^a^* estimates based on survey generalized linear regression models assuming a Gaussian distribution and a log link function.

^b^ estimates based on survey multinomial logit models (relative risk ratios reported; RRRs).

CRP: C-Reactive Protein. STAI-10: Spielberger Trait Anxiety Inventory

*Crude*: unadjusted.

*Age & Sex Adj*: Crude + age, sex

*Dem Adj:* Age & Sex Adj + Latino background, education, income, marital status.

*Risk Adj:* Dem Adj *+* BMI, systolic BP, diastolic BP, current smoker, alcohol drinks/week.

*Med Adj:* Risk Adj*+* antidepressants, antianxiety agents, antihypertensives, hypoglycemic, lipid lowering medications.

***p<0.001 **p<0.01 *p<0.05

**Supp Figure 1.** Estimated marginal means of the hsCRP levels. The increases in circulating hsCRP levels over the range of (a) CES-D and (b) STAI scores and their 95% confidence intervals are plotted from the crude (M1) and fully-adjusted models (M5), controlling for sociodemographic (age, sex, Hispanic/Latino background, education, marital status, and income), health and behavior (BMI, systolic and diastolic BP, current smoker, alcohol drinks/week), and medications (antidepressants, antianxiety agents, antihypertensives, hypoglycemic, lipid lowering medications).


**Supplemental Figure 2.** Estimated marginal probabilities for the hsCRP risk categories. The estimated probabilities for each hsCRP risk group (low, intermediate, and high) over the range of CES-D (top) and STAI (bottom) scores and their 95% confidence intervals are plotted from the crude (M1) and fully-adjusted models (M5), controlling for sociodemographic (age, sex, Hispanic/Latino background, education, marital status, and income), health and behavior (BMI, systolic and diastolic BP, current smoker, alcohol drinks/week), and medications (antidepressants, antianxiety agents, antihypertensives, hypoglycemic, lipid lowering medications)
